# Effect of sodium–glucose cotransporter 2 inhibitors on cardiac structure and function in type 2 diabetes mellitus patients with or without chronic heart failure: a meta-analysis

**DOI:** 10.1186/s12933-020-01209-y

**Published:** 2021-01-25

**Authors:** Yi-Wen Yu, Xue-Mei Zhao, Yun-Hong Wang, Qiong Zhou, Yan Huang, Mei Zhai, Jian Zhang

**Affiliations:** State Key Laboratory of Cardiovascular Disease, Heart Failure Center, National Center for Cardiovascular Diseases, Fuwai Hospital, Chinese Academy of Medical Sciences and Peking Union Medical College, Beijing, People’s Republic of China

**Keywords:** Sodium–glucose cotransporter 2 inhibitors, Type 2 diabetes mellitus, Chronic heart failure, Cardiac remodelling

## Abstract

**Background:**

Although the benefits of sodium–glucose cotransporter 2 inhibitors (SGLT2i) on cardiovascular events have been reported in patients with type 2 diabetes mellitus (T2DM) with or without heart failure (HF), the impact of SGLT2i on cardiac remodelling remains to be established.

**Methods:**

We searched the PubMed, Embase, Cochrane Library and Web of Science databases up to November 16th, 2020, for randomized controlled trials reporting the effects of SGLT2i on parameters of cardiac structure, cardiac function, plasma N-terminal pro-brain natriuretic peptide (NT-proBNP) level or the Kansas City Cardiomyopathy Questionnaire (KCCQ) score in T2DM patients with or without chronic HF. The effect size was expressed as the mean difference (MD) or standardized mean difference (SMD) and its 95% confidence interval (CI). Subgroup analyses were performed based on the stage A–B or stage C HF population and HF types.

**Results:**

Compared to placebo or other antidiabetic drugs, SGLT2i showed no significant effects on left ventricular mass index, left ventricular end diastolic volume index, left ventricular end systolic volume index, or left atrial volume index. SGLT2i improved left ventricular ejection fraction only in the subgroup of HF patients with reduced ejection fraction (MD 3.16%, 95% CI 0.11 to 6.22, p = 0.04; I^2^ = 0%), and did not affect the global longitudinal strain in the overall analysis including stage A–B HF patients. SGLT2i showed benefits in the E/e’ ratio (MD − 0.45, 95% CI − 0.88 to − 0.03, p = 0.04; I^2^ = 0%), plasma NT-proBNP level (SMD − 0.09, 95% CI − 0.16 to − 0.03, p = 0.004; I^2^ = 0%), and the KCCQ score (SMD 3.12, 95% CI 0.76 to 5.47, p  = 0.01; I^2^ = 0%) in the overall population.

**Conclusion:**

The use of SGLT2i was associated with significant improvements in cardiac diastolic function, plasma NT-proBNP level, and the KCCQ score in T2DM patients with or without chronic HF, but did not significantly affect cardiac structural parameters indexed by body surface area. The LVEF level was improved only in HF patients with reduced ejection fraction.

## Background

Heart failure (HF) is one of the leading causes of morbidity and mortality worldwide. Type 2 diabetes mellitus (T2DM) can cause diabetic cardiomyopathy, which typically manifests first as left ventricular hypertrophy, diastolic dysfunction, and impaired systolic reserve before gradually showing clinical indications of heart failure with preserved ejection fraction (HFpEF), followed by systolic dysfunction and heart failure with reduced ejection fraction (HFrEF) [1]. T2DM also increases the risk of coronary heart disease and subsequent HF, especially HFrEF [2]. Besides, both in HFrEF and HFpEF patients, comorbid T2DM is associated with a worse prognosis [3–5].

The effects of sodium–glucose cotransporter 2 inhibitors (SGLT2i) on the prognosis (including all-cause death, cardiovascular death, and HF hospitalization) of T2DM [6–9] patients with or without HF [10–12] have been demonstrated in large-scale randomized controlled trials (RCTs) and meta-analyses. Based on clinical evidence, SGLT2i was recommended by the latest guidelines of the American Diabetes Association and the European Association for the Study of Diabetes in patients with T2DM and HF [13], and several agents were recommended by the Heart Failure Association of the European Society of Cardiology in T2DM patients at high cardiovascular risk or with established cardiovascular disease, especially symptomatic HFrEF [14]. However, the mechanism and intermediate links of the drugs remain to be clarified.

Cardiac anatomical and functional parameters partially predict the prognosis and quality of life of patients with T2DM and patients with HF and serve as important surrogate endpoints. Experiments in rodent T2DM models revealed the benefits of SGLT2i on left ventricular hypertrophy [15] and dilation [16], as well as cardiac systolic [15] and diastolic functions [15, 17]. In rodent and porcine nondiabetic HFrEF models, SGLT2i improved left ventricular ejection fraction (LVEF) [18–20] but not diastolic function [20], and showed conflicting results in left ventricular structure [18–21]. In animal models of HFpEF with or without T2DM, SGLT2i improved left ventricular structure [22] and diastolic function [22, 23], but did not affect LVEF [23].

Recent clinical studies have also reported conflicting results. In T2DM patients, the DAPA-LVH trial showed that SGLT2i reversed left ventricular hypertrophy compared to placebo [24], but the EMPA-HEART CardioLink-6 trial showed nonsignificant results [25]. The impacts on LVEF [25, 26], global longitudinal strain (GLS) [24, 27], and diastolic function [25, 28] were also inconsistent in different studies. Similarly, in patients with T2DM and HF, the effects of SGLT2i on left ventricular hypertrophy [27, 29], cardiac function [27, 30, 31], and neurohormonal parameters [32, 33] were inconsistent. Whether such diversity was due to insufficient sample size or heterogeneity among studies remains to be explored.

To make better use of up-to-date clinical evidence, we conducted this meta-analysis to further clarify the effect of SGLT2i on cardiac structure, cardiac function, plasma N-terminal pro-brain natriuretic peptide (NT-proBNP) level and the Kansas City Cardiomyopathy Questionnaire (KCCQ) score in T2DM patients with or without chronic HF. Subgroup analyses were performed based on the stage A–B or stage C HF population and HF types.

## Methods

This meta-analysis was conducted following the Preferred Reporting Items for Systematic Reviews and Meta-Analyses (PRISMA) guidelines [34].

### Search strategy and selection criteria

**Fig. 1:**
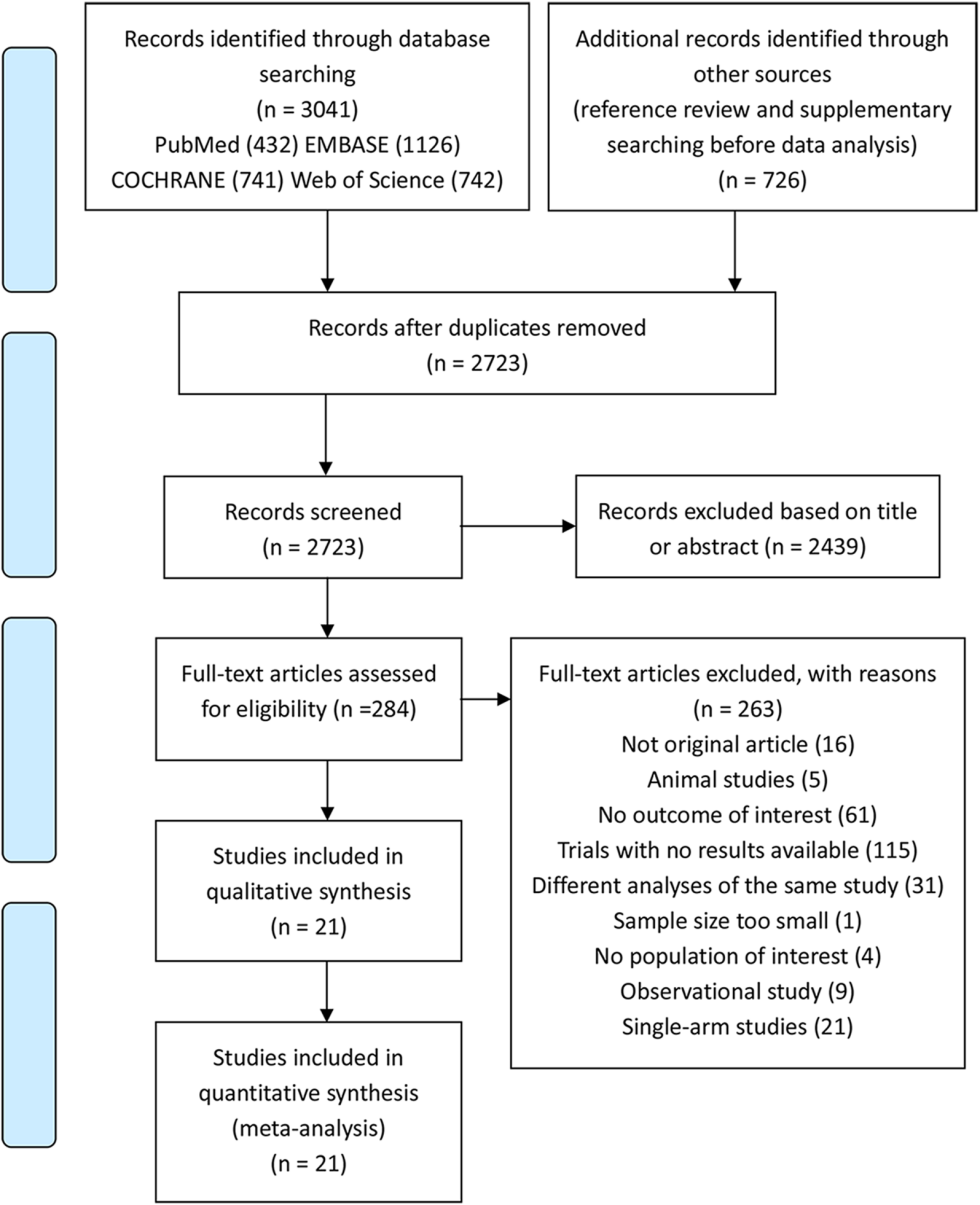
PRISMA flow diagram of study selection

We systematically searched PubMed, Embase, Cochrane Library and Web of Science databases up to November 16th, 2020, using specific MeSH terms and random words with no restriction of language or publication status. The inclusion criteria were as follows: (1) reported the effect of SGLT2i in adult T2DM patients (≥ 18 years) with or without chronic HF; (2) placebo or other antidiabetic agents were accepted as comparison; (3) reported the outcomes of interest; (4) was an RCT; and (5) had complete data for extraction. Observational studies, single-arm studies, studies in acute heart failure patients and studies with a sample size of < 10 were excluded. The reference lists of eligible studies and related articles were reviewed manually to identify additional studies. The main search was conducted on April 21st, 2020, and the supplementary search was performed before data analysis with the same strategy in case of omission. We also sent data request letters by email to the authors of articles with insufficient data for analysis. In the case of two independent reports of the same study, only the one with more complete data was included. Searching details and the flow diagram (including the exclusion criteria) are available in Additional file 1: Data S1 and Fig. 1.

### Data extraction

The extracted data included (1) general information: title, author, publication year, trial name, eligibility and the reasons; (2) clinical information: age, sex, country or area of the participants; specific agent of the SGLT2 inhibitor given to the experiment group; therapy for the control group; whether the participant was diagnosed as HF at baseline; HF types by reduced or preserved ejection fraction; (3) data for overall effect size calculation: the sample size of each group, as well as the mean value and standard deviation (SD) of the change of outcomes before and after treatment in each group; and (4) methodological information. Data were extracted from the main article reporting the included studies, related articles reporting the same study, and the study registry websites. T2DM patients with an established diagnosis of HF was classified as stage C HF, and those without were classified as stage A–B HF.

### Quality assessment of eligible studies

We used the revised Cochrane risk-of-bias tool to assess the quality of the RCTs (see Additional file 2: Figure S1). Publication bias was evaluated by funnel plots (see Additional file 3: Figure S2). Egger’s regression asymmetry test was conducted to assess the significance of funnel plot asymmetries.

We assessed the certainty of the evidence for each outcome using the Grading Recommendations Assessment, Development and Evaluation (GRADE) approach. We used the Guideline Development Tool (https://www.gradepro.org) to formulate the evidence profile table.

Literature search, study selection, data extraction, quality assessment of eligible studies and the GRADE assessment were performed by two researchers (YWY and YHW) independently, and disagreements were resolved by consensus.

### Outcomes

The outcomes of this meta-analysis were (1) cardiac anatomic changes including left ventricular mass indexed by body surface area (LVMI), left ventricular end-diastolic volume indexed by body surface area (LVEDVI), left ventricular end-systolic volume indexed by body surface area (LVESVI), and left atrial volume indexed by body surface area (LAVI); (2) cardiac functional changes including LVEF, GLS, and the mitral inflow to mitral relaxation velocity ratio (E/eʹ); (3) changes in plasma NT-proBNP level; and (4) the KCCQ score, or the score of any scale in the questionnaire including the symptom section.

### Data analysis

All the variables of interest were continuous and expressed as the mean ± SD. Data reported as the median and interquartile range were transformed to the mean and SD according to the methods suggested by McGrath [35] and Wan [36]. The SD was calculated according to the Cochrane Handbook [37] if results were reported in other forms [p values or confidence intervals (CI)]. The NT-proBNP level reported as the geometric means or geometric mean ratio and 95% CI in three studies were converted to log-transformed scale and analyzed by the generic inverse variance method [38], as sensitivity analysis for the studies reported in the raw scale. The KCCQ score was also analyzed by the generic inverse variance method due to incomplete reporting of the mean ± SD in each group. We used a random-effects model for all the analyses. The effects of SGLT2i on the outcomes were compared between the intervention and comparison arms. Pooled results were expressed as the mean difference (MD) or standardized mean difference (SMD) and its 95% CI. A two-sided P < 0.05 was considered significant. The heterogeneity of the results was assessed using I^2^ statistics. Sensitivity analyses included heterogeneity analysis using the leave-one-out method, analysis of only high-quality studies, and analysis of only studies using placebo as the control group. Subgroup analyses were performed if each subgroup contains two or more studies, basing on the stage A–B or stage C HF population and the LVEF level in stage C HF patients. All analyses were performed using Review Manager software version 5.4 (The Cochrane Collaboration), R version 3.6.1 (R Foundation for Statistical Computing), and STATA software version 15.0 (StataCorp LP, College Station, TX, USA).

## Results

A total of 21 RCTs [10, 24, 25, 29–31, 33, 39–52] were recognized eligible in this meta-analysis, including 3 in crossover design [41, 44, 50]. A total of 10,978 participants were enrolled, including 6236 in the SGLT2i group and 4821 in the control group. Seventy percent of the participants were male, and the mean age ranged from 56 to 73 years old. The mean follow-up period ranged from 14 days to one year, including three studies [41, 50, 51] less than 3 months. Participants with T2DM that were mostly in stage A–B HF were enrolled in 10 studies [24, 25, 39, 40, 44, 46, 48, 49, 51, 52], and patients with T2DM and stage C HF were enrolled in 11 studies [10, 29–31, 33, 41–43, 45, 47, 50]. LVMI, LVEDVI, LVESVI, LAVI, LVEF, GLS, the E/e’ ratio, plasma NT-proBNP level, and the KCCQ score were reported in 6 [24, 25, 29, 44, 45, 52], 3 [25, 29, 30], 3 [25, 29, 30], 4 [25, 29, 45, 52], 9 [24, 25, 29–31, 39, 40, 44, 45], 4 [24, 39, 44, 51], 8 [24, 25, 30, 31, 44, 45, 49, 52], 11 [10, 24, 25, 31, 41, 44–46, 48–50] and 3 [10, 42, 43] studies, respectively. Cardiac structure and function were evaluated by magnetic resonance imaging in 4 studies [24, 25, 29, 51], echocardiography in 8 studies [24, 30, 31, 39, 44, 45, 49, 52], and impedance cardiography in 1 study [40]. The treatment for the control group was placebo in 15 studies [10, 24, 25, 29, 33, 40–44, 46, 47, 50–52], and conventional treatment or other antidiabetic drugs in 6 studies [30, 31, 39, 45, 48, 49]. Baseline characteristics of the eligible studies were presented in Table 1.

**Table 1:** Baseline characteristics of eligible studies

Author	Publication year	Study design	Trial	Country/area	Intervention in treatment and control arms	Population	Overall sample size (n)	Follow-up period	Age (year, mean ± SD)	Sex (male%)	Imaging	Parameters
Januzzi	2017	RCT	–	Multiple countries and areas	Canagliflozin 100 mg/day or 300 mg/day; placebo	Older patients with T2DM	666	52 weeks	63.74 ± 6.31	57.27%	–	NT-proBNP
Bonora	2019	RCT	DAPA-HDL	Italy	Dapagliflozin 10 mg/day; placebo	T2DM, excluding HF patients with NYHA classes III-IV	30	12 weeks	63.4 ± 6.9	66.70%	ICG	LVEF
Brown	2020	RCT	DAPA-LVH	UK	Dapagliflozin 10 mg/day; placebo	T2DM, excluding patients diagnosed as clinical HF	66	12 months	65.53 ± 6.87	57.60%	MRI; ECHO	LVMI, LVEF, GLS, E/e', NT-proBNP
Ikonomidis	2020	RCT	–	Greece	SGLT2i; standard care without SGLT2i	T2DM	160	12 months	58 ± 10	72%	ECHO	LVEF, GLS
Katakami	2020	RCT	UTOPIA	Japan	Tofogliflozin 20 mg/day; conventional drugs	T2DM	340	52 weeks	61.10 ± 9.49	58.40%	–	NT-proBNP
Kayano	2020	RCT	–	Japan	Dapagliflozin 5 mg/day; conventional therapy	T2DM candidates with hypertension (grade 1 or 2) and/or a history of ischemic heart disease	74	6 months	67.65 ± 8.53	89.18%	ECHO	E/eʹ, NT-proBNP
Oldgren	2020	RCT	DAPACARD	Sweden and Finland	Dapagliflozin 10 mg/day; placebo	T2DM with normal left ventricular ejection fraction (≥ 50%) assessed within 1 year	49	6 weeks	64.4 ± 7.2	53%	MRI	GLS
Shim	2020	RCT	IDDIA	Korea	Dapagliflozin 10 mg/day; placebo	T2DM and LV diastolic dysfunction	60	24 weeks	–	–	ECHO	LVMI, LAVI, E/eʹ
Verma	2019	RCT	EMPA-HEART CardioLink-6	Canada	Empagliflozin 10 mg/day; placebo	T2DM and CAD, excluding patients with an LVEF < 30%, NYHA class IV or hospitalized for decompensated HF within the preceding 3 months	97	6 months	67.6 ± 6.6	80%	MRI	LVMI, LVEDVI, LVESVI, LAVI, LVEF, E/eʹ, NT-proBNP
Anker	2020	RCT	EMPEROR-Reduced	Multiple countries and areas	Empagliflozin 10 mg/day; placebo	T2DM and CHF	1856	52 weeks	66.70 ± 10.15	76.90%	–	KCCQ, NT-proBNP
Bhatt	2020	RCT	SOLOIST-WHF	Multiple countries and areas	Sotagliflozin 200 mg/day; placebo	T2DM recently hospitalized for worsening heart failure	1222	4 months	69.90 ± 9.34	66.24%	–	KCCQ
Carbone	2020	RCT	CANA-HF	US	Canagliflozin 100 mg/day; sitagliptin 100 mg/day	T2DM and HFrEF	36	12 weeks	56.1 ± 7.8	77.77%	ECHO	LVEDVI, LVESVI, LVEF, E/eʹ
de Boer	2020	RCT	–	Multiple countries and areas	Empagliflozin 25 mg/day; placebo	T2DM and CHF	63	12 weeks	68.02 ± 9.10	61.93%	–	NT-proBNP
Eickhoff	2020	RCT (crossover)	DapKid	Denmark	Dapagliflozin 10 mg/day; placebo	T2DM and CHF	40	12 weeks	64 ± 8	89%	ECHO	LVMI, LVEF, GLS, E/eʹ, NT-proBNP
Ejiri	2020	RCT	MUSCAT-HF	Japan	Luseogliflozin 2.5 mg/day; voglibose	T2DM and HFpEF	165	12 weeks	73.1412 ± 7.8130	62.52%	ECHO	LVMI, LAVI, LVEF, E/eʹ, NT-proBNP
Griffin	2020	RCT (crossover)	–	US	Empagliflozin 10 mg/day; placebo	T2DM and HF	20	14 days	60 ± 12	75%	–	NT-proBNP
Januzzi	2020	RCT	CANVAS; CANVAS-R	Multiple countries and areas	Canagliflozin 100 or 300 mg/day; placebo	T2DM and high risk for cardiovascular events	3587	1 year	62.6999 ± 7.8670	67%	–	NT-proBNP
Mordi	2020	RCT (crossover)	RECEDE-CHF	UK	Empagliflozin 25 mg/day; placebo	T2DM and CHF	23	6 weeks	69.8 ± 5.7	73.90%	–	NT-proBNP
Petrie	2020	RCT	DAPA-HF	Multiple countries and areas	Dapagliflozin 10 mg/day; placebo	T2DM and HFrEF	2139	8 months	66.50 ± 9.85	77.70%	–	NT-proBNP, KCCQ
Singh	2020	RCT	REFORM	UK	Dapagliflozin 10 mg/day; placebo	T2DM and CHF	56	1 year	67.1	66.10%	MRI	LVMI, LVEDVI, LVESVI, LAVI, LVEF
Tanaka	2020	RCT	CANDLE	Japan	Canagliflozin 100 mg/day; glimepiride 0.5 to 6.0 mg/day	T2DM and HF	233	24 weeks	68.6 ± 10.1	74.71%	ECHO	LVEF, E/e', NT-proBNP

*RCT* randomized controlled trial, *SGLT2i* sodium–glucose cotransporter 2 inhibitors, *T2DM* type 2 diabetes mellitus, *HF* heart failure, *CHF* chronic heart failure, *HFrEF* heart failure with reduced ejection fraction, *HFpEF* heart failure with preserved ejection fraction, *LVEF* left ventricular ejection fraction, *CAD* coronary artery disease, *CV* cardiovascular, *NYHA* New York Heart Association, *MRI* magnetic resonance imaging, *ECHO* echocardiography, *ICG* impedance cardiography, *LVMI* left ventricular mass indexed by body surface area, *LVEDVI* left ventricular end diastolic volume indexed by body surface area, *LVESVI* left ventricular end systolic volume indexed by body surface area, *LAVI* left atrial volume indexed by body surface area, *GLS* global longitudinal strain: *E/e'* mitral inflow to mitral relaxation velocity ratio, *NT-proBNP* N-terminal pro-brain natriuretic peptide, *KCCQ* the Kansas City Cardiomyopathy Questionnaire, *UK* the United Kingdom, *US* the United States

### Results of the main analyses and sensitivity analyses

**Fig. 2:**
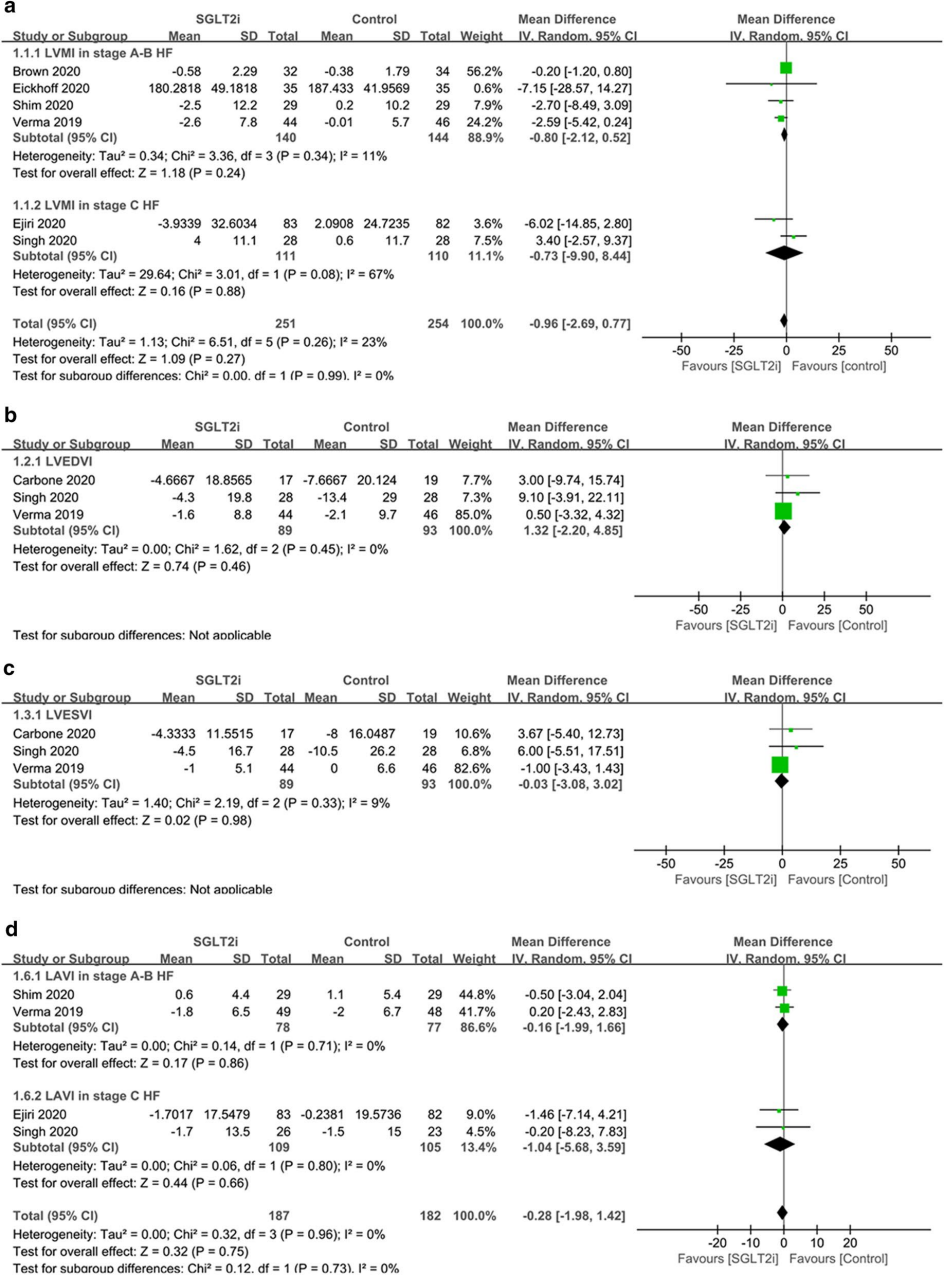
Forest plots of the effects of SGLT2i on cardiac structure indexed by body surface area. **a** LVMI; **b** LVEDVI; **c** LVESVI; **d** LAVI. *SGLT2i* sodium–glucose cotransporter 2 inhibitors, *T2DM* type 2 diabetes mellitus, *HF* heart failure, *LVMI* left ventricular mass indexed by body surface area, *LVEDVI* left ventricular end diastolic volume indexed by body surface area, *LVESVI* left ventricular end systolic volume indexed by body surface area, *LAVI* left atrial volume indexed by body surface area

The use of SGLT2i showed no significant effect on LVMI compared with placebo or other antidiabetic drugs in T2DM patients with or without HF (MD -0.96 g/m^2^, 95% CI − 2.69 to 0.77, p = 0.27; I^2^ = 23%) (Fig. 2). LVEDVI, LVESVI and LAVI were also not significantly changed by the use of SGLT2i compared to the control group in the overall population (MD 1.32 ml/m^2^, 95% CI −2.20 to 4.85, p = 0.46; I^2^ = 0%; MD −.03 ml/m^2^, 95% CI −3.08 to 3.02, p = 0.98; I^2^ = 9%; MD −.28 ml/m^2^, 95% CI − 1.98 to 1.42, p = 0.75; I^2^ = 0%) (Fig. 2).

**Fig. 3:**
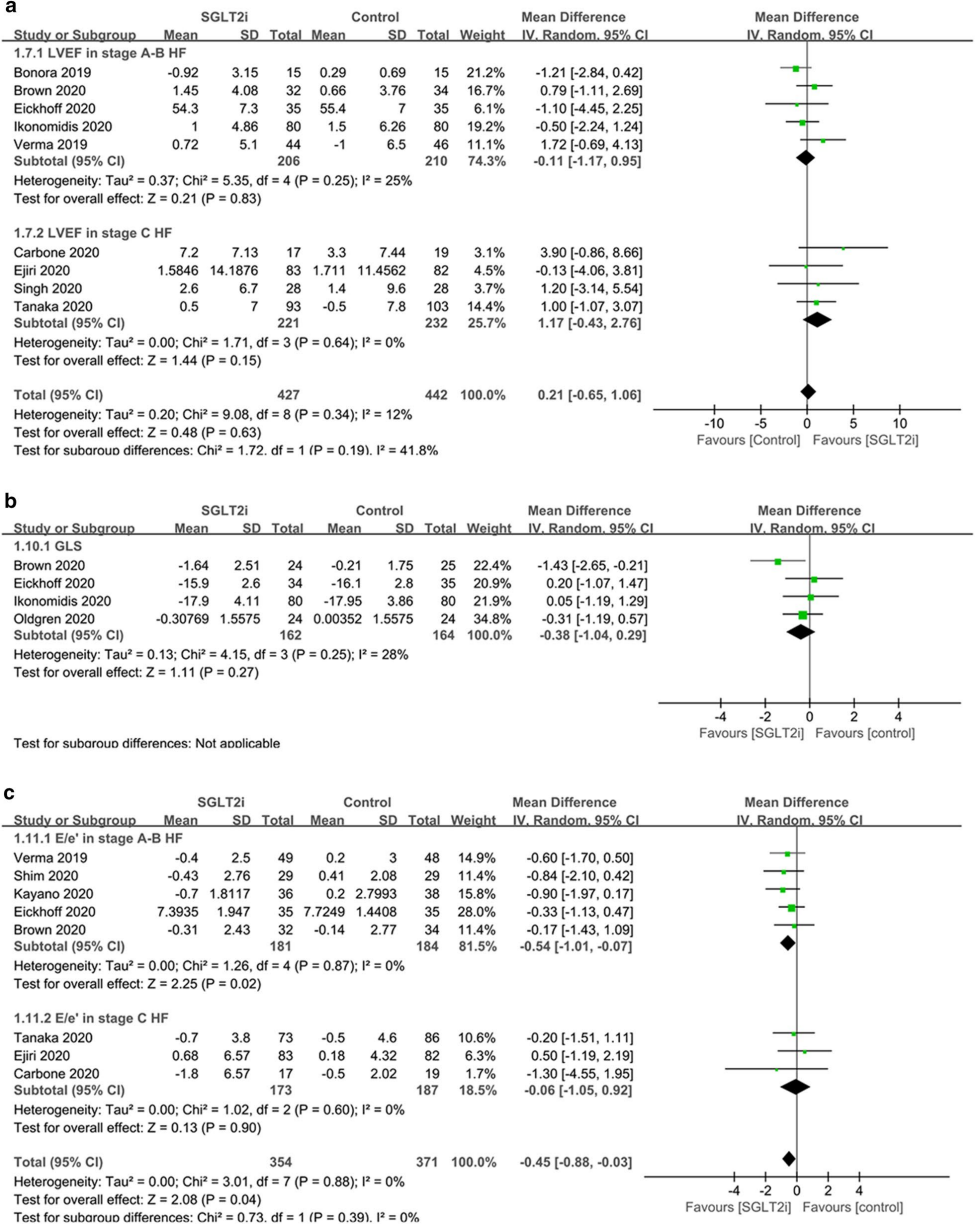
Forest plots of the effects of SGLT2i on cardiac function. **a** LVEF; **b** GLS; **c** E/eʹ. *SGLT2i* sodium–glucose cotransporter 2 inhibitors, *T2DM* type 2 diabetes mellitus, *HF* heart failure, *LVEF* left ventricular ejection fraction, *GLS* global longitudinal strain, *E/eʹ* mitral inflow to mitral relaxation velocity ratio

As for systolic function, SGLT2i did not have a significant effect on LVEF (MD 0.21%, 95% CI − 0.65 to 1.06, p = 0.63; I^2^ = 12%) (Fig. 3) or GLS (MD − 0.38%, 95% CI − 1.04 to 0.29, p = 0.27; I^2^ = 28%) (Fig. 3) in the overall population. For left ventricular diastolic function, the use of SGLT2i was associated with a reduction of the E/eʹ ratio (MD − 0.45, 95% CI − 0.88 to − 0.03, p = 0.04; I^2^ = 0%) (Fig. 3). Sensitivity analysis including only the 7 high-quality studies showed a similar reduction of the E/eʹ ratio by SGLT2i, and analysis including the 4 placebo-controlled studies showed insignificant results.

**Fig. 4:**
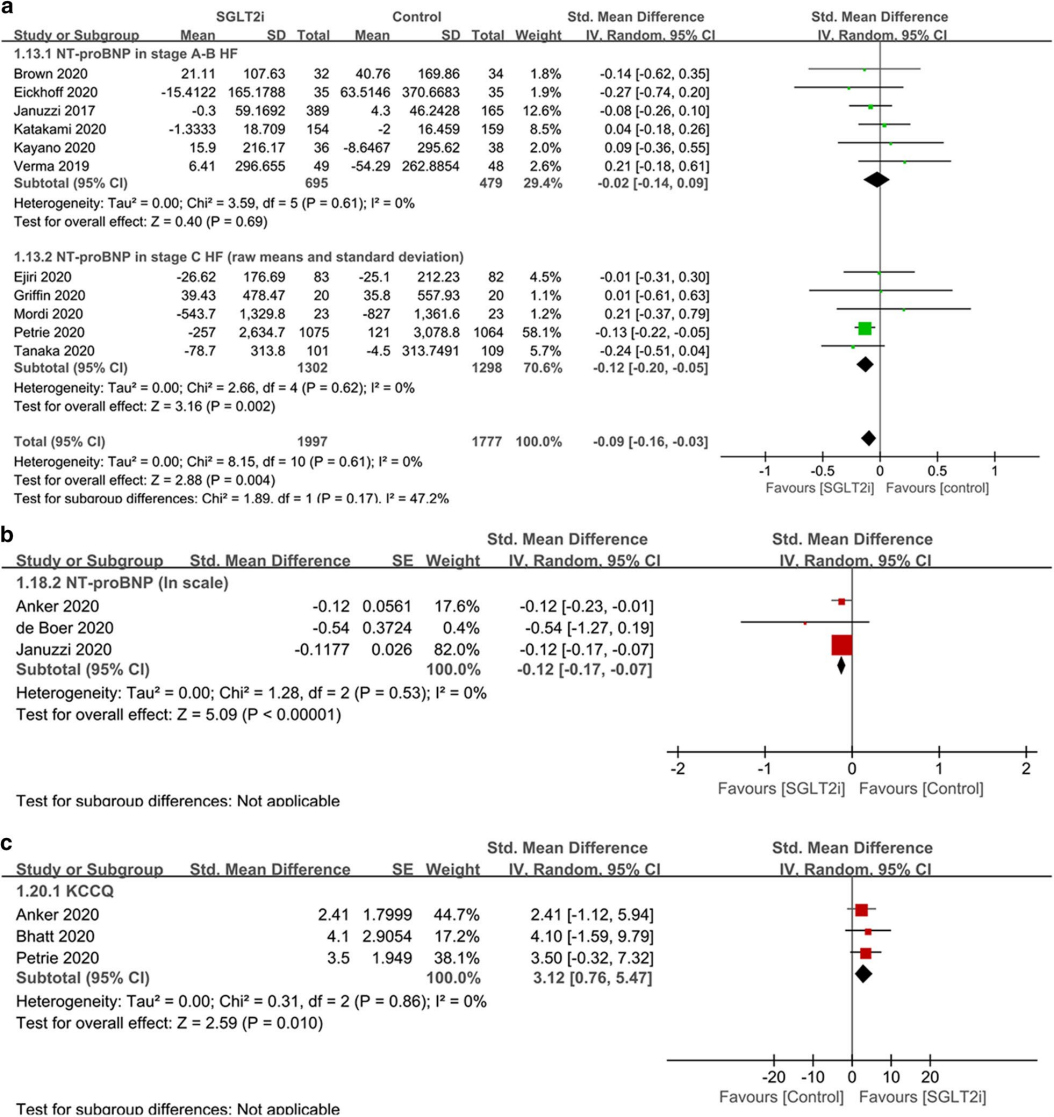
Forest plots of the effects of SGLT2i on **a** NT-proBNP and **b** KCCQ score. *SGLT2i* sodium–glucose cotransporter 2 inhibitors, *T2DM* type 2 diabetes mellitus, *HF* heart failure, *NT-proBNP* N-terminal pro-brain natriuretic peptide, *KCCQ* the Kansas City Cardiomyopathy Questionnaire

The use of SGLT2i reduced the plasma NT-proBNP levels (SMD − 0.09, 95% CI − 0.16 to − 0.03, p = 0.004; I^2^ = 0%) (Fig. 4) in the overall population. The three studies reporting data in the geometric scales could not be pooled with those reporting data in the raw scale, thus served as sensitivity analysis, and showed consistent results as in the main analysis (SMD − 0.12, 95% CI − 0.17 to − 0.07, p < 0.00001; I^2^ = 0%) (Fig. 4). Other sensitivity analyses included only the 9 high quality studies and only the 7 placebo-controlled studies, both showed consistent results with the main analysis.

The KCCQ score was significantly improved by SGLT2i compared with placebo or other antidiabetic drugs (SMD 3.12, 95% CI 0.76 to 5.47, p = 0.01; I^2^ = 0%) (Fig. 4). The KCCQ items used were different among the three eligible trials, including the total symptom score in the DAPA-HF trial, the total symptom score and physical limitation score in the EMPEROR-Reduced trial, and the KCCQ-12 items score in the SOLOIST-WHF trial. All the trials were placebo-controlled and of high quality, so sensitivity analysis was not conducted.

### Results of subgroup analyses

Subgroup analyses of LVMI and LAVI based on stage A–B or stage C HF population showed insignificant results. We did not conduct subgroup analysis in LVEDVI and LVESVI because only three studies reported the outcomes.

LVEF was not significantly changed by the use of SGLT2i compared to placebo or other antidiabetic drugs in subgroup analysis based on stage A–B or stage C HF population. Nevertheless, in subgroup analyses in stage C HF patients based on HF types, SGLT2i was related to improved LVEF in HFrEF patients (MD 3.16%, 95% CI 0.11 to 6.22, p = 0.04; I^2^ = 0%), but was insignificant in HFpEF patients (MD 0.19%, 95% CI − 1.76 to 2.15, p = 0.85; I^2^ = 0%) (Additional file 4: Figure S3). All the studies reporting GLS were in stage A–B HF patients with T2DM, so subgroup analysis was not conducted. SGLT2i improved the E/e’ ratio in stage A–B HF population (MD − 0.54, 95% CI − 1.01 to − 0.07, p = 0.02; I^2^ = 0%) but not in stage C HF population (MD − 0.06, 95% CI − 1.05 to 0.92, p = 0.9; I^2^ = 0%) (Fig. 3). In stage C HF patients, SGLT2i did not significantly affect the E/e’ ratio in both the HFrEF (MD − 0.33, 95% CI − 2.76 to 2.10, p = 0.79; I^2^ = 0%) and HFpEF (MD − 0.19, 95% CI − 1.23 to 0.85, p = 0.72; I^2^ = 2%) groups (Additional file 4: Figure S3).

The use of SGLT2i reduced the NT-proBNP level in stage C HF population (SMD − 0.12, 95% CI − 0.20 to − 0.05, p = 0.002; I^2^ = 0%) but not in stage A–B HF population (SMD − 0.02, 95% CI − 0.14 to 0.09, p = 0.69; I^2^ = 0%) (Fig. 4). In stage C HF patients, SGLT2i significantly improved the NT-proBNP level in the HFrEF subgroup (SMD − 0.14, 95% CI − 0.22 to − 0.05, p = 0.001; I^2^ = 0%) but not in the HFpEF subgroup (SMD − 0.07, 95% CI − 0.29 to 0.14, p = 0.51; I^2^ = 0%) (Additional file 4: Figure S3).

All the three studies reporting the KCCQ score were conducted in stage C HF patients with T2DM and subgroup analysis was not performed.

### Quality assessment and publication bias

Quality assessments of each of the RCTs are shown in Additional file 2: Figure S1. Among the 21 RCTs included in this meta-analysis, 14 were considered to be at low risk, 3 with some concerns, and 4 were at high risk, which was mainly driven by the open-label design in the studies by Tanaka et al*.* and Katakami et al*.*, and the high missing rate in the studies by Ikonomidis et al. and de Boer et al. The results of publication bias assessment are shown in Additional file 3: Figure S2. According to the results of Egger’s asymmetry test, there was no obvious publication bias in any of the analyses (p > 0.05).

According to the GRADE evidence profile (Table 2), the certainty of the evidence was moderate for most of the outcomes, except for LVEF in HFrEF population, which showed a low certainty mostly driven by the high risk of bias in the study by Tanaka et al*.*; and for the KCCQ score, which showed a high certainty.

**Table 2:** GRADE evidence profile

Certainty assessment							No. of patients		Effect		Certainty	Importance
No. of studies	Study design	Risk of bias	Inconsistency	Indirectness	Imprecision	Other considerations	SGLT2i	Control	Relative (95% CI)	Absolute (95% CI)		
LVMI												
6	Randomized trials	Not serious	Not serious	Not serious	Serious^a^	None	251	254	–	MD *0.96 lower* (2.69 lower to 0.77 higher)	⨁⨁⨁◯ Moderate	Important
LVEDVI												
3	Randomized trials	Not serious	Not serious	Not serious	Serious^a^	None	89	93	–	MD *1.32 higher* (2.20 lower to 4.85 higher)	⨁⨁⨁◯ Moderate	Important
LVESVI												
3	Randomized trials	Not serious	Not serious	Not serious	Serious^a^	None	89	93	–	MD *0.03 lower* (3.08 lower to 3.02 higher)	⨁⨁⨁◯ Moderate	Important
LAVI												
4	Randomized trials	Not serious	Not serious	Not serious	Serious^a^	None	187	182	–	MD *0.28 lower* (1.98 lower to 1.42 higher)	⨁⨁⨁◯ Moderate	Important
LVEF												
9	Randomized trials	Not serious	Not serious	Not serious	Serious^a^	None	427	442	-	MD *0.21 higher* (0.65 lower to 1.06 higher)	⨁⨁⨁◯ Moderate	Important
LVEF in HFrEF												
2	Randomized trials	Serious ^b^	Not serious	Not serious	Serious^a^	None	70	74	–	MD *3.16 higher* (0.11 higher to 6.22 higher)	⨁⨁◯◯ Low	Important
LVEF in HFpEF												
2	Randomized trials	Not serious	Not serious	Not serious	Serious^a^	None	151	158	–	MD *0.19 higher* (1.76 lower to 2.15 higher)	⨁⨁⨁◯ Moderate	Important
GLS												
4	Randomized trials	Not serious	Not serious	Not serious	Serious^a^	None	162	164	–	MD *0.38 lower* (1.04 lower to 0.29 higher)	⨁⨁⨁◯ Moderate	Important
E/eʹ												
8	Randomized trials	Not serious	Not serious	Not serious	Serious^a^	None	354	371	–	MD *0.45 lower* (0.88 lower to 0.03 lower)	⨁⨁⨁◯ Moderate	Important
NT-proBNP												
11	Randomized trials	Not serious	Not serious	Not serious	Serious^a^	None	1997	1777	–	SMD *0.09 lower *(0.16 lower to 0.03 lower)	⨁⨁⨁◯ Moderate	Critical
NT-proBNP (ln scale)												
3	Randomized trials	Not serious	Not serious	Not serious	Serious^a^	None	2702	1629	–	SMD *0.12 lower* (0.17 lower to 0.07 lower)	⨁⨁⨁◯ Moderate	Critical
KCCQ												
3	Randomized trials	Not serious	Not serious	Not serious	Not serious	None	2610	2607	–	SMD *3.12 higher* (0.76 higher to 5.47 higher)	⨁⨁⨁⨁ High	Critical

*SGLT2i* sodium–glucose cotransporter 2 inhibitors, *MD* mean difference, *SMD* standardized mean difference, *HFrEF* heart failure with reduced ejection fraction, *HFpEF* heart failure with preserved ejection fraction, *LVMI* left ventricular mass indexed by body surface area, *LVEDVI* left ventricular end diastolic volume indexed by body surface area, *LVESVI* left ventricular end systolic volume indexed by body surface area, *LAVI* left atrial volume indexed by body surface area, *LVEF* left ventricular ejection fraction, *GLS* global longitudinal strain, *E/e'* mitral inflow to mitral relaxation velocity ratio, *NT-proBNP* N-terminal pro-brain natriuretic peptide, *KCCQ* the Kansas City Cardiomyopathy Questionnaire

^a^Sample size below optimal information size contributing to imprecision which lowers our certainty in effect

^b^One in the two studies is of high risk according to the Cochrane risk of bias tool. And excluding the study would cause change of the results

## Discussion

This meta-analysis comprehensively and quantitively analyses the effects of SGLT2i on cardiac structure, cardiac function, plasma NT-proBNP level and the KCCQ score in T2DM patients with or without chronic HF. The main findings of this study included the following: (1) SGLT2i showed no significant effects on LVMI, LVEDVI, LVESVI, and LAVI; (2) SGLT2i improved LVEF in HFrEF patients but not in HFpEF patients or stage A–B HF patients with T2DM, and showed no significant effects on GLS in stage A–B HF patients with T2DM; (3) SGLT2i reduced the E/e’ ratio in the overall population and stage A–B HF patients but not in stage C HF patients; (4) SGLT2i improved the plasma NT-proBNP level in the overall population and stage C HF patients, and showed no significant results in stage A–B HF patients; and (5) SGLT2i improved the KCCQ score in stage C HF patients with T2DM.

Our searching and analysis results on the LVM, LVEDV, and LVESV were the same as those reported in a recently published meta-analysis [53], thus were not presented in this article. Pooled analysis of two studies [24, 25] reporting LVM measured by MRI in stage A–B HF population showed a significant reduction after the use of SGLT2i compared to placebo or other antidiabetic drugs (MD − 3.04 g, 95% CI − 5.14 to − 0.94, p = 0.005; I^2^ = 0%). The inconsistency in the results of SGLT2i regarding LVM and LVMI may be attributed to the concomitant effect of weight loss, which was also observed in studies included in our analysis and a previous meta-analysis [24, 29, 31, 39, 54]. Since LVMI was calculated by LVM indexed by body surface area (BSA), which was influenced by both temporal height and weight of the individual, weight loss would obscure the estimation of the actual anatomical change of the heart. This was previously discussed in the study by Brown et al*.* [24], showing that SGLT2i significantly reduced LVM as well as LVM indexed by height or baseline BSA but not that indexed by real-time BSA. LVM was demonstrated to be a risk factor for the decline of LVEF [55] as well as all-cause and cardiovascular mortality [56] in stage A–B HF. The decrease of LVM might be related to the reduction of the incidence of stage C HF observed in previous RCTs. Despite larger sample sizes than the studies reporting LVM, the use of SGLT2i showed no significant effects on LVEDV, LVESV, LVEDVI, LVESVI, and LAVI, suggesting a null or faint effect of the drug on the dilation of cardiac chambers. Since the increase of LVM usually reflects both enlargement of the left ventricle and thickening of the walls, the results above may imply an effect of SGLT2i on the wall thickness rather than on the ventricle volume, which is to be demonstrated in future studies.

Taken together, the results of the overall and subgroup analyses suggested that SGLT2i significantly reduced LVEF in HFrEF patients but not in HFpEF patients, showing benefits in patients with obvious systolic dysfunction. However, the results in HFrEF subgroup suffered from a low certainty in the GRADE evidence profile, calling for more future studies in the population. The effect of SGLT2i on GLS, a more sensitive parameter reflecting even mild systolic dysfunction [57–59], was not significant in the pooled analysis. Nevertheless, GLS was reported in four RCTs in stage A–B HF patients with T2DM but not yet in stage C HF patients. Ongoing trials such as ERTU-GLS (NCT03717194) in the T2DM and stage C HF population would provide more evidence. As for diastolic dysfunction, the E/eʹ ratio was reduced by SGLT2i in the overall population and stage A–B HF patients, but not in stage C HF patients. The discrepancy between the subgroups could be due to the mild and more reversible impairment of the diastolic dysfunction in stage A–B HF patients, whereas large-scale trials are still needed.

The use of SGLT2i significantly reduced the plasma NT-proBNP level in the stage C HF population. However, the effect on NT-proBNP level between the SGLT2i and control group was − 333 pg/ml in the T2DM subgroup in the DAPA-HF trial [10] (median baseline level in the SGLT2i group: 1479 pg/ml), and − 103 pg/ml in the whole population of EMPEROR-Reduced trial [42] (median baseline level in the SGLT2i group: 1894 pg/ml) declaring no significant difference in patients with and without T2DM. Those changes were moderate and inconsistent with the remarkable influence of SGLT2i on the cardiovascular events [60], suggesting that NT-proBNP could not be considered to be a satisfying surrogate endpoint for efficacy assessment in this case.

In the pre-SGLT2i age, the change of NT-proBNP level used to be expected to predict the effect size of HF therapy on cardiovascular outcomes. One meta-analysis [61] suggested a significant association between changes in NT-proBNP level and the risk of hospital stay for HF worsening. In the PARADIGM-HF trial [62], the use of angiotensin receptor-neprilysin inhibitor in HFrEF patients induced a 30% decline in NT-proBNP level after the run-in period of 4–6 weeks, and the reduction was associated with the change in cardiovascular mortality and HF hospitalization rate. However, the relationship was less strong in the PARAGON-HF trial [63] in HFpEF patients, which showed a considerable effect of SGLT2i on the reduction of NT-proBNP but a moderate effect on the primary outcome in the subgroups of men and patients with higher LVEF. Moreover, the termination of the GUIDE-IT trial [64] due to futility suggested against the add-on NT-proBNP-guided strategy versus guideline-directed medical therapy alone in HFrEF patients. Updated evidence from the trials in SGLT2i further supported the view that the NT-proBNP could not be used generally as a predictor of the hard endpoints, but may be indicative for specific drugs or in certain subgroups of patients.

Pooled results of the three large-scale RCTs reporting the KCCQ score showed significant improvement by SGLT2i compared with placebo in T2DM patients with stage C HF. As for the magnitude of the effect, analysis of the T2DM subgroup in DAPA-HF trial [10] showed that more patients reported an increase of at least 5 points in the SGLT2i group compared with the placebo group (58.9% vs 49.9%), yielding a number needed to treat of 14 patients with dapagliflozin for one to be clinically better in eight months, which showed a considerable benefit [65]. The MD in the change of the KCCQ score was 4.1 points (95% CI 1.3 to 7.0) in the SOLOIST-WHF trial and 2.41 (95% CI 0.64 to 4.17) in the T2DM subgroup in EMPEROR-Reduced trial, but the numbers needed to treat were not calculable. The benefit on symptoms and quality of life associated with SGLT2i was consistent with the noteworthy reduction in the risk of hospitalization for heart failure in the T2DM subgroup of the DAPA-HF and EMPEROR-Reduced trials.

Despite the clinically significant improvement of quality of life and cardiovascular outcomes by SGLT2i, the debate on the underlying mechanism of the drug is still on the way. The most known mechanism of SGLT2i is based on excess excretion of fluid and glucose and modest removal of sodium [66]. Diuresis alleviates cardiac preload, leading to reduced blood pressure [67], left ventricular wall stress, and left ventricular filling pressure. This could be the reason for the reduction of the NT-proBNP level and the E/e’ ratio that we have observed. However, the significant effect of SGLT2i on LVM but not ventricular volume could not be fully interpreted by the theory above. Other possible mechanisms such as more efficient energy source of ketone bodies and fatty acids rather than glucose [19, 68], relieving inflammation [69, 70], and reducing fibrosis and oxidative stress [15], may also play a role. The previously prompted hypothesis of the inhibition of cardiac Na+–H+ Exchanger-1 was however challenged in a recent in vitro study [71]. Still, further research is required to illuminate the complete picture.

Previous systemic and narrative reviews [72–75] summarized completed and ongoing studies available on the same topic as ours. However, they were mostly conducted before the releasing of results of several important recent studies and thus lacked sufficient data for quantitative analyses. This meta-analysis included only RCTs but not observational studies to minimize the possible risk of bias, and used the GRADE tool to assess the certainty of the evidence for each outcome. Although conducted strictly following the PRISMA guidelines, the meta-analysis still has some limitations. First, in subgroup analyses, we stratified the T2DM population as stage A–B and stage C HF patients. But in some studies recognized as stage A–B HF, HF patients were not fully excluded. Second, heterogeneity in clinical characteristics and study methods was not completely avoidable, in consideration of which we used a random-effects model for all the analyses. Third, subgroup analyses based on the dosage forms of SGLT2i and the modality of imaging were not conducted due to insufficient data, which remain to be clarified in future studies.

Large-scale RCTs focusing on the effects of SGLT2i in different populations are required to provide more evidence for individualized intervention. The results of the ongoing EMPA-TROPISM (NCT03485222) [76], EMPA-HEART (EUDRACT 2016-0022250-10) [77], ERTU-GLS (NCT03717194), NATRIURETIC (NCT04535960), VERTICAL (NCT04490681), EMPERIAL-Preserved and EMPERIAL-Reduced (NCT03448406, NCT03448419) [78] trials would enhance knowledge of this topic. Although the efficacy and safety of SGLT2i in several dosage forms have been repeatedly verified in T2DM patients with or without HF to support the clinical application, the underlying mechanism remains to be clarified to achieve a more comprehensive understanding.

## Conclusion

We found in this meta-analysis that SGLT2i improves the parameters of cardiac diastolic function, plasma NT-proBNP level, and the KCCQ score in T2DM patients with or without chronic HF, but did not significantly affect cardiac structural parameters indexed by body surface area. The LVEF level was improved only in HF patients with reduced ejection fraction. Future studies are anticipated to further elucidate the mechanisms and intermediate links in the effect of SGLT2i.

## Supplementary Information

**Additional file 1: Data S1.** Strategy for the main search conducted on April 21^st^, 2020.

**Additional file 2: Figure S1.** Quality assessment of RCTs using the revised Cochrane risk-of-bias tool. (a) Risk of bias graph; (b) Risk of bias summary.

**Additional file 3: Figure S2.** Funnel plots for publication bias assessment. (a) LVMI; (b) LVEDVI; (c) LVESVI; (d) LAVI; (e) LVEF; (f) GLS; (g) E/e’; (h) NT-proBNP; (i) KCCQ.

**Additional file 4: Figure S3.** Subgroup analyses of the effects of SGLT2i on (a) LVEF in HFrEF vs. HFpEF patients; (b) E/e’ in HFrEF vs. HFpEF patients; (c) NT-proBNP in HFrEF vs. HFpEF patients. Abbreviations: SGLT2i: sodium-glucose cotransporter 2 inhibitors; HFrEF: heart failure with reduced ejection fraction; HFpEF: heart failure with preserved ejection fraction; LVEF: left ventricular ejection fraction; E/e': mitral inflow to mitral relaxation velocity ratio; NT-proBNP: N-terminal pro-brain natriuretic peptide.

## Data Availability

Not applicable.

## Abbreviations

HF: Heart failure
T2DM: Type 2 diabetes mellitus
HFpEF: Heart failure with preserved ejection fraction
HFrEF: Heart failure with reduced ejection fraction
SGLT2i: Sodium–glucose cotransporter 2 inhibitors
RCT: Randomized controlled trial
LVEF: Left ventricular ejection fraction
GLS: Global longitudinal strain
NT-proBNP: N-terminal pro-brain natriuretic peptide
KCCQ: The Kansas City Cardiomyopathy Questionnaire
PRISMA: The Preferred Reporting Items for Systematic Reviews and Meta-Analyses
SD: Standard deviation
GRADE: The Grading Recommendations Assessment, Development and Evaluation
LVMI: Left ventricular mass indexed by body surface area
LVEDVI: Left ventricular end diastolic volume indexed by body surface area
LVESVI: Left ventricular end systolic volume indexed by body surface area
LAVI: Left atrial volume indexed by body surface area
E/e': The mitral inflow to mitral relaxation velocity ratio
CI: Confidence interval
MD: Mean difference
SMD: Standardized mean difference
BSA: Body surface area
