# Supplementary material for: Effect of sodium–glucose cotransporter 2 inhibitors on cardiac structure and function in type 2 diabetes mellitus patients with or without chronic heart failure: a meta-analysis
Source: Cardiovasc Diabetol. 2021 Jan 25;20:25. doi: 10.1186/s12933-020-01209-y (PMC7836497; doi:10.1186/s12933-020-01209-y)
Supplement: Supplementary file 1 — Additional file 1: Data S1. Strategy for the main search conducted on April 21st, 2020. [file 12933_2020_1209_MOESM1_ESM.pdf]

PubMed search strategy:

((((((((("heart"[MeSH Terms] OR "heart"[Title/Abstract]) OR "cardiac"[Title/Abstract]) OR "myocardial"[Title/Abstract]) OR "myocardiac"[Title/Abstract]) OR "diabetes"[Title/Abstract]) OR "hypertension"[Title/Abstract]) OR "high blood pressure"[Title/Abstract]) AND (((((((((((((("sodium-glucose transporter 2 inhibitors"[MeSH Terms] OR "sodium glucose transporter 2 inhibitors"[Title/Abstract]) OR "sglt2 inhibitor"[Title/Abstract]) OR "sglt 2 inhibitors"[Title/Abstract]) OR "sglt 2 inhibitors"[Title/Abstract]) OR "Gliflozins"[Title/Abstract]) OR "sodium glucose cotransporter 2 inhibitor"[Title/Abstract]) OR "sodium glucose cotransporter 2 inhibitor"[Title/Abstract]) OR "empagliflozin"[Title/Abstract]) OR "canagliflozin"[Title/Abstract]) OR "dapagliflozin"[Title/Abstract]) OR "luseogliflozin"[Title/Abstract]) OR "tofogliflozin"[Title/Abstract]) OR "sotagliflozin"[Title/Abstract]) OR "ertugliflozin"[Title/Abstract]) OR "ipragliflozin"[Title/Abstract])) AND (((((((((("function"[Title/Abstract] OR "dysfunction"[Title/Abstract]) OR "ejection fraction"[Title/Abstract]) OR "remodeling"[Title/Abstract]) OR "left ventricle"[Title/Abstract]) OR "left ventricular"[Title/Abstract]) OR "left atrium"[Title/Abstract]) OR "left atrial"[Title/Abstract]) OR "quality of life"[Title/Abstract]) OR "qol"[Title/Abstract]) OR "fibrosis"[Title/Abstract])) NOT ("animals"[MeSH Terms] NOT "humans"[MeSH Terms])) NOT (("Review"[Publication Type] OR "meta-analysis"[Publication Type]))

Translations

heart[MeSH Terms]: "heart"[MeSH Terms]

Sodium-Glucose Transporter 2 Inhibitors[MeSH Terms]: "sodium-glucose transporter 2 inhibitors"[MeSH Terms]

animals[MeSH Terms]: "animals"[MeSH Terms]

humans[MeSH Terms]: "humans"[MeSH Terms]

Embase search strategy:

('heart'/exp OR heart OR cardiac:ab,ti OR myocardial:ab,ti OR myocardiac:ab,ti OR diabetes:ab,ti OR hypertension:ab,ti OR 'high blood pressure':ab,ti) AND ('sodium glucose cotransporter 2 inhibitor'/exp OR 'sodium glucose cotransporter 2 inhibitor' OR (('sodium'/exp OR sodium) AND ('glucose'/exp OR glucose) AND ('cotransporter'/exp OR cotransporter) AND 2 AND ('inhibitor'/exp OR inhibitor)) OR 'sglt2 inhibitor':ab,ti OR 'sglt-2 inhibitors':ab,ti OR 'sglt 2 inhibitors':ab,ti OR gliflozins:ab,ti OR 'sodium glucose cotransporter 2 inhibitor':ab,ti OR 'sodium-glucose cotransporter 2 inhibitor':ab,ti OR empagliflozin:ab,ti OR canagliflozin:ab,ti OR dapagliflozin:ab,ti OR luseogliflozin:ab,ti OR tofogliflozin:ab,ti OR sotagliflozin:ab,ti OR ertugliflozin:ab,ti OR ipragliflozin:ab,ti) AND (function:ab,ti OR dysfunction:ab,ti OR 'ejection fraction':ab,ti OR remodeling:ab,ti OR 'left ventricle':ab,ti OR 'left ventricular':ab,ti OR 'left atrium':ab,ti OR 'left atrial':ab,ti OR 'quality of life':ab,ti OR qol:ab,ti OR fibrosis:ab,ti) NOT ('animal'/exp NOT 'human'/exp) NOT (review:it OR editorial:it OR 'meta analysis':it)

The Cochrane Library search strategy:

ID Search Hits

#1 MeSH descriptor: [Heart] explode all trees 6715

#2 (heart):ti,ab,kw OR (cardiac):ti,ab,kw OR (myocardial):ti,ab,kw OR (myocardiac):ti,ab,kw OR (diabetes):ti,ab,kw (Word variations have been searched) 244480

#3 (hypertension):ti,ab,kw OR (high blood pressure):ti,ab,kw (Word variations have been

searched) 78152
#4 #1 OR #2 OR #3 286501
#5 MeSH descriptor: [Sodium-Glucose Transporter 2 Inhibitors] explode all trees 231
#6 (Sodium-Glucose Transporter 2 Inhibitors):ti,ab,kw OR (sglt2 inhibitor):ti,ab,kw OR (SGLT-2 Inhibitors):ti,ab,kw OR (SGLT 2 Inhibitors):ti,ab,kw OR (Gliflozins):ti,ab,kw (Word variations have been searched) 1261
#7 (sodium glucose cotransporter 2 inhibitor):ti,ab,kw OR (sodium-glucose cotransporter 2 inhibitor):ti,ab,kw OR (empagliflozin):ti,ab,kw OR (canagliflozin):ti,ab,kw OR (dapagliflozin):ti,ab,kw (Word variations have been searched) 2440
#8 (luseogliflozin):ti,ab,kw OR (tofogliflozin):ti,ab,kw OR (sotagliflozin):ti,ab,kw OR (ertugliflozin):ti,ab,kw OR (ipragliflozin):ti,ab,kw (Word variations have been searched) 456
#9 #5 OR #6 OR #7 OR #8 2849
#10 (function):ti,ab,kw OR (dysfunction):ti,ab,kw OR (ejection fraction):ti,ab,kw OR (remodeling):ti,ab,kw OR (left ventricle):ti,ab,kw (Word variations have been searched) 273887
#11 (left ventricular):ti,ab,kw OR (left atrium):ti,ab,kw OR (left atrial):ti,ab,kw OR (quality of life):ti,ab,kw OR (qol):ti,ab,kw (Word variations have been searched) 130127
#12 (fibrosis):ti,ab,kw (Word variations have been searched) 13061
#13 #10 OR #11 OR #12 354624
#14 MeSH descriptor: [Animals] explode all trees 30512
#15 MeSH descriptor: [Humans] explode all trees 23698
#16 #14 NOT #15 6814
#17 #4 AND #9 AND #13 NOT #16 741

Web of science search strategy:

# 5 742 #1 AND #2 AND #3 NOT #4
Indexes=SCI-EXPANDED, CPCI-S Timespan=All years
# 4 371,100 (TS=(function) OR TS=(dysfunction) OR TS=(ejection fraction) OR TS=(remodeling) OR TS=(left ventricle) OR TS=(left ventricular) OR TS=(left atrium) OR TS=(left atrial) OR TS=(quality of life) OR TS=(qol) OR TS=(fibrosis)) AND DOCUMENT TYPES: (Review)
Indexes=SCI-EXPANDED, CPCI-S Timespan=All years
# 3 5,390,702 TOPIC: (function) OR TOPIC: (dysfunction) OR TOPIC: (ejection fraction) OR TOPIC: (remodeling) OR TOPIC: (left ventricle) OR TOPIC: (left ventricular) OR TOPIC: (left atrium) OR TOPIC: (left atrial) OR TOPIC: (quality of life) OR TOPIC: (qol) OR TOPIC: (fibrosis)
Indexes=SCI-EXPANDED, CPCI-S Timespan=All years
# 2 6,560 TOPIC: (Sodium-Glucose Transporter 2 Inhibitors) OR TOPIC: (sglt2 inhibitor) OR TOPIC: (SGLT-2 Inhibitors) OR TOPIC: (SGLT 2 Inhibitors) OR TOPIC: (Gliflozins) OR TOPIC: (sodium glucose cotransporter 2 inhibitor) OR TOPIC: (sodium-glucose cotransporter 2 inhibitor) OR TOPIC: (empagliflozin) OR TOPIC: (canagliflozin) OR TOPIC: (dapagliflozin) OR TOPIC: (luseogliflozin) OR TOPIC: (tofogliflozin) OR TOPIC: (sotagliflozin) OR TOPIC: (ertugliflozin) OR TOPIC: (ipragliflozin)
Indexes=SCI-EXPANDED, CPCI-S Timespan=All years
# 1 2,279,415 TOPIC: (heart) OR TOPIC: (cardiac) OR TOPIC: (myocardial) OR TOPIC: (myocardiac) OR TOPIC: (diabetes) OR TOPIC: (hypertension) OR TOPIC: (high blood pressure)
Indexes=SCI-EXPANDED, CPCI-S Timespan=All years
