# Supplementary figures and images for: Effect of sodium–glucose cotransporter 2 inhibitors on cardiac structure and function in type 2 diabetes mellitus patients with or without chronic heart failure: a meta-analysis
Source: Cardiovasc Diabetol. 2021 Jan 25;20:25. doi: 10.1186/s12933-020-01209-y (PMC7836497; doi:10.1186/s12933-020-01209-y)

a.

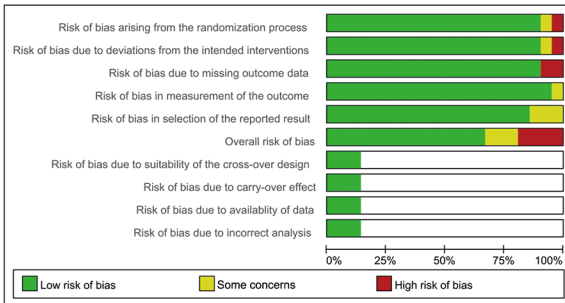

b.

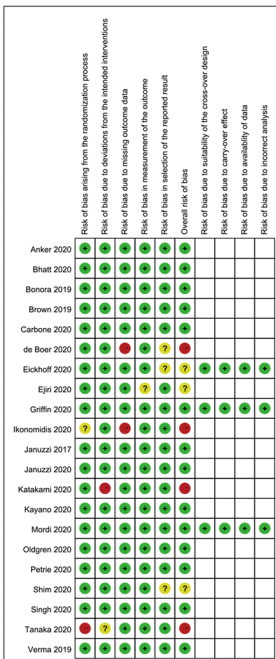

Supplement: Supplementary file 2 — Additional file 2: Figure S1. Quality assessment of RCTs using the revised Cochrane risk-of-bias tool. (a) Risk of bias graph; (b) Risk of bias summary. [file 12933_2020_1209_MOESM2_ESM.pdf]

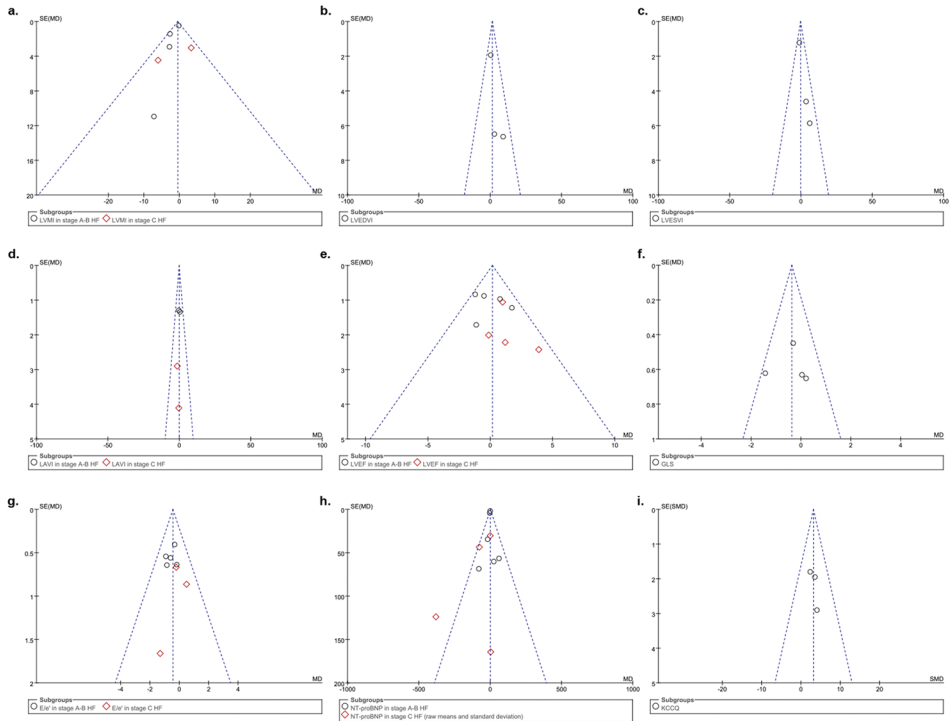

Supplement: Supplementary file 3 — Additional file 3: Figure S2. Funnel plots for publication bias assessment. (a) LVMI; (b) LVEDVI; (c) LVESVI; (d) LAVI; (e) LVEF; (f) GLS; (g) E/e’; (h) NT-proBNP; (i) KCCQ. [file 12933_2020_1209_MOESM3_ESM.pdf]

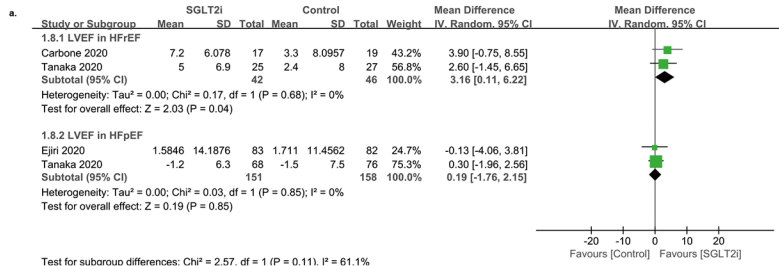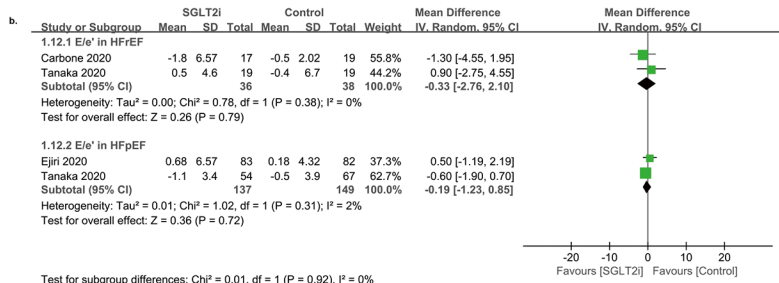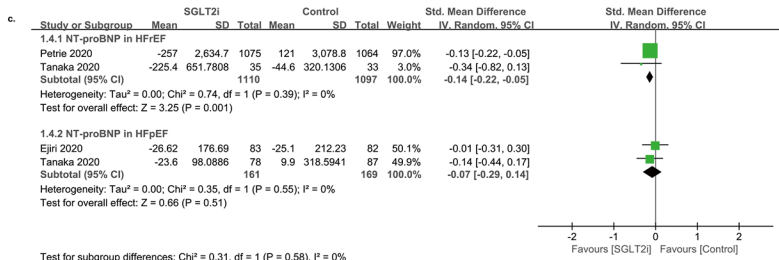

Supplement: Supplementary file 4 — Additional file 4: Figure S3. Subgroup analyses of the effects of SGLT2i on (a) LVEF in HFrEF vs. HFpEF patients; (b) E/e’ in HFrEF vs. HFpEF patients; (c) NT-proBNP in HFrEF vs. HFpEF patients. Abbreviations: SGLT2i: sodium-glucose cotransporter 2 inhibitors; HFrEF: heart failure with reduced ejection fraction; HFpEF: heart failure with preserved ejection fraction; LVEF: left ventricular ejection fraction; E/e': mitral inflow to mitral relaxation velocity ratio; NT-proBNP: N-terminal pro-brain natriuretic peptide. [file 12933_2020_1209_MOESM4_ESM.pdf]
